# Does multimorbidity result in de-prioritisation of COPD in primary care?

**DOI:** 10.1038/s41533-023-00326-x

**Published:** 2023-01-14

**Authors:** Carolina Smith, Mikael Hasselgren, Christer Janson, Marta A. Kisiel, Karin Lisspers, Anna Nager, Hanna Sandelowsky, Björn Ställberg, Josefin Sundh, Scott Montgomery

**Affiliations:** 1 School of Medical Sciences, Faculty of Medicine and Health, Örebro University, Örebro, Sweden; 2 Centre for Clinical Research and Education, Region Värmland, Karlstad, Sweden; 3 Department of Medical Sciences, Respiratory, Allergy and Sleep Research, Uppsala University, Uppsala, Sweden; 4 Department of Medical Sciences, Occupational and Environment Medicine, Uppsala University, Uppsala, Sweden; 5 Department of Public Health and Caring Sciences, Family Medicine and Preventive Medicine, Uppsala University, Uppsala, Sweden; 6 NVS, Division of Family Medicine and Primary Care, Karolinska Institutet, Stockholm, Sweden; 7 Clinical Epidemiology Division, Department of Medicine, Solna, Karolinska Institutet, Stockholm, Sweden; 8 Department of Neurobiology, Care Sciences and Society, Division of Family Medicine and Primary Care, Karolinska Institutet, Stockholm, Sweden; 9 Academic Primary Health Care Centre, Region Stockholm, Stockholm, Sweden; 10 Department of Respiratory Medicine, Faculty of Medicine and Health, Örebro University, Örebro, Sweden; 11 Clinical Epidemiology and Biostatistics, School of Medical Sciences, Faculty of Medicine and Health, Örebro University, Örebro, Sweden; 12 Department of Epidemiology and Public Health, University College London, London, UK

**Keywords:** Chronic obstructive pulmonary disease, Epidemiology

## Abstract

The aim of this study was to describe factors associated with having COPD regularly reviewed in primary care by a nurse or physician and assess whether there was de-prioritisation for COPD in multimorbid patients. We defined de-prioritisation as not having at least one check-up by a physician during a two-year period. Among 713 COPD patients in the Swedish PRAXIS study, 473 (66%) had at least one check-up during the study period (ending in 2014). Patients with check-ups were more likely to have three or more comorbid conditions (31.9% vs. 24.6%) and exacerbations (35.1% vs. 21.7%) than those without. Compared with those without comorbidity, those with three or more diagnoses had increased relative risk ratios (and 95% CI) for consultations discussing COPD with only a physician (5.63 (2.68–11.79)), COPD-nurse only (1.67 (0.83–3.37)) or both (2.11 (1.09–4.06)). COPD patients received more frequent check-ups considering COPD if they had comorbidity or a history of exacerbations. We found no evidence of de-prioritisation for COPD in multimorbid patients.

## Introduction

Chronic obstructive pulmonary disease (COPD) frequently coexists with other long-term conditions^1^. Multimorbidity, defined as having at least two long-term conditions, is thus common in COPD patients^2^ and known to increase both COPD-related morbidity and mortality^3^. COPD is itself an important comorbidity that could adversely influence the outcome of other disorders^1^. Management of chronic diseases is often based on clinical guidelines with a focus on single diseases, only occasionally taking the patient’s broader disease burden into account^4^. Therefore, long-term management of patients with COPD can be a comprehensive, time-consuming, and complex task.

In Sweden, most COPD patients are managed in primary health care with annual check-ups by a general practitioner (GP). During these check-ups, the patient’s health status is evaluated, and prescriptions are renewed, often for the next 12 months. Swedish GPs regularly manage multiple health conditions during a single consultation, which is why such a visit can take up to 30 min or longer. However, a recent study has suggested that GPs de-prioritise the management of COPD in multimorbid patients because of time constraints^5^.

Most GPs in Sweden are employed by primary health care centres (PHCCs). Due to a shortage of GPs, the health care centres often employ locum GPs on short-term contracts. Most PHCCs have a nurse-led COPD clinic, where a specialist primary care nurse performs spirometry, supports self-management and reviews COPD patients. These clinics are integrated into the PHCC and have been found to improve COPD outcomes^6^. The patient does not need a referral from the GP to see the nurse. The nurse-led check-ups focus on COPD management, and rarely consider comorbidity. Thus, a visit to the nurse is not a replacement for a check-up by the GP for the multimorbid patient, but an addition. If the patient has severe COPD and needs specialised treatment, such as long-term oxygen therapy, the GP can refer the patient to specialist hospital care.

The aims of this study were: (1) to describe patient- and caregiver-related factors associated with having routine check-ups for COPD in primary care by a GP, COPD nurse or both, and (2) to assess whether there is evidence of de-prioritisation of COPD in multimorbid patients compared to patients with no comorbidity. De-prioritisation of COPD was defined as not receiving routine check-ups by a GP where COPD was noted in the medical records at least once during a two-year period.

## Results

**Fig. 1:**
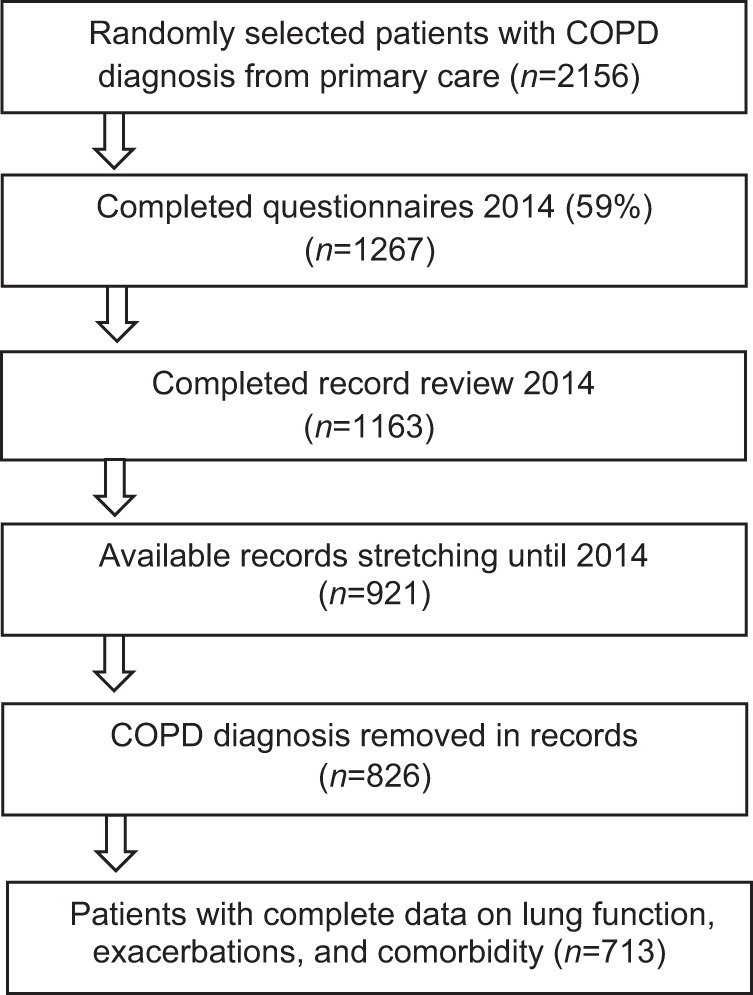
Flow chart. Selection of patients eligible for inclusion in the analysis.

**Fig. 2:**
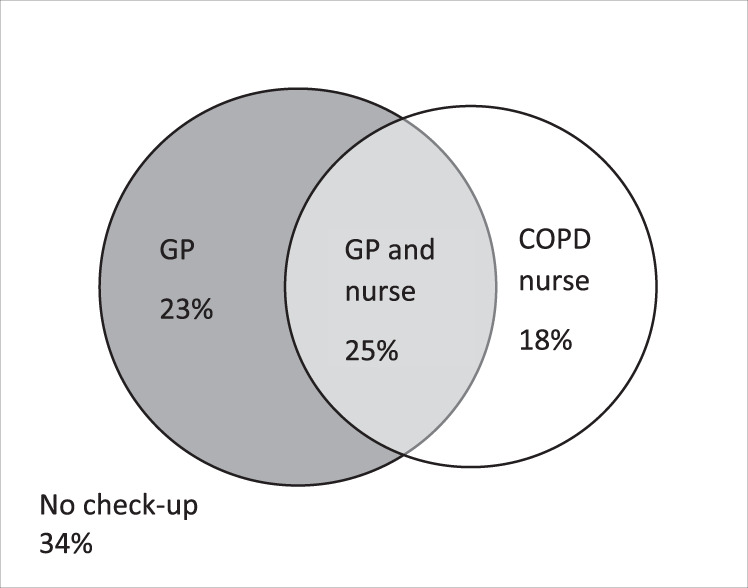
Check-ups by general practitioner, COPD nurse, or both. Venn diagram showing the proportion of the study population having at least one check-up during the two-year period by a general practitioner (GP), COPD nurse or both.

A total of 713 COPD patients with complete data were included in the analysis (Fig. 1). The characteristics of the patients with and without routine check-ups are shown in Table 1. Some 34% of the patients had no routine check-ups and 66% had at least one during the two-year period (see Fig. 2). The patients in the two groups had similar distributions of age, sex, and body mass index (BMI). Patients who had check-ups had lower FEV_1_% of predicted, more frequent exacerbations, more often had cardiovascular comorbidity and a higher number of comorbid conditions than those without check-ups.

**Table 1:** Patient characteristics.

*N* = 713	*N* (%)	Routine check-ups by GP, COPD nurse, or both		
		None *N* = 240 (33.7)	≥1 *N* = 473 (66.3)	*p*-value
*Sex*				0.865
Female	386 (54.1)	131 (54.6)	255 (53.9)	
Male	327 (45.9)	109 (45.4)	218 (46.1)	
*Age*, *years*				
≤60	105 (14.7)	38 (15.8)	67 (14.2)	Ref
61–65	106 (14.9)	37 (15.4)	69 (14.6)	0.845
66–70	223 (31.3)	80 (33.3)	143 (30.2)	0.956
71–75	240 (33.7)	71 (29.6)	169 (35.7)	0.224
>75	39 (5.5)	14 (5.8)	25 (5.3)	0.974
*BMI*				
<18.5	30 (4.2)	10 (4.2)	20 (4.2)	0.805
18.5–24.9	264 (37.0)	94 (39.2)	170 (35.9)	Ref
25–29.9	248 (34.8)	82 (34.2)	166 (35.1)	0.545
≥30	171 (24.0)	54 (22.5)	117 (24.7)	0.387
*FEV*_*1*_*%pred*				
≥80	105 (14.7)	45 (18.8)	60 (12.7)	Ref
50–79	402 (56.4)	139 (57.9)	263 (55.6)	0.116
30–49	164 (23.0)	43 (17.9)	121 (25.6)	0.005
<30	42 (5.9)	13 (5.4)	29 (6.1)	0.182
*Comorbidity*				
Heart failure	78 (10.9)	16 (6.7)	62 (13.1)	0.009
IHD	112 (15.7)	30 (12.5)	82 (17.3)	0.094
Atrial fibrillation	63 (8.8)	16 (6.7)	47 (9.9)	0.146
Hypertension	404 (56.7)	118 (49.2)	286 (60.5)	0.004
Stroke/TIA	55 (7.7)	16 (6.7)	39 (8.2)	0.455
Diabetes	141 (19.8)	40 (16.7)	101 (21.4)	0.138
Anxiety	112 (15.7)	43 (17.9)	69 (14.6)	0.248
Depression	150 (21.0)	46 (19.2)	104 (22.0)	0.383
Osteoporosis	54 (7.6)	17 (7.1)	37 (7.8)	0.724
Other cancer	80 (11.2)	22 (9.2)	58 (12.3)	0.216
Asthma	73 (10.2)	27 (11.3)	46 (9.7)	0.526
*Exacerbation*^**a**^	218 (30.6)	52 (21.7)	166 (35.1)	<0.001
*Number of comorbid conditions*				
0	115 (16.1)	58 (24.2)	57 (12.1)	Ref
1	214 (30.0)	67 (27.9)	147 (31.1)	0.001
2	174 (24.4)	56 (23.3)	118 (24.9)	0.002
≥3	210 (29.5)	59 (24.6)	151 (31.9)	<0.001

Patient characteristics distributed over having had check-ups by GP, COPD nurse, or both, at least once in the previous 2 years.

*GP* general practitioner, *BMI* Body Mass Index, *FEV1%pred* FEV1% of predicted, *IHD* ischaemic heart disease, *TIA* transient ischaemic attack.

^a^Exacerbation ≥1 previous 6 months.

Among all patients, 84% had one or more other long-term conditions, 29% had three or more. Hypertension was the most common comorbidity (57%), followed by depression (21%) and diabetes (20%).

The multinomial logistic regression analysis with check-ups (no check-up, only by GP, only by COPD nurse, or by both GP and COPD nurse) as the dependent variable, and no check-up as the reference category, showed statistically significant associations with check-ups only by the GP for exacerbations (relative risk ratio 2.64, 95% confidence interval 1.61–4.35) and hypertension (RRR 2.41, 95% CI 1.49–3.88). Positive associations for heart failure, ischaemic heart disease, and diabetes were seen in the unadjusted analyses but did not remain statistically significant after adjustment. When using the comorbidity count instead of individual comorbid disorders in the model, there were higher relative risk ratios for a larger number of diseases (see Table 2).

**Table 2:** Factors associated with having check-ups by a general practitioner, COPD nurse, or both.

	Check-up only by GP, *n* = 166 (23.3%)					Check-up only by COPD nurse, *n* *=* *128 (17.9%)*					Check-up by both GP and COPD nurse, *n* = 179 (25.1%)				
	*N* (%)	Unadjusted		Adjusted		*N* (%)	Unadjusted		Adjusted		*N* (%)	Unadjusted		Adjusted	
		RRR (95% CI)	*p*-value	RRR (95% CI)	*p*-value		RRR (95% CI)	*p*-value	RRR (95% CI)	*p*-value		RRR (95% CI)	*p*-value	RRR (95% CI)	*p*-value
*Age, years*															
≤60	23 (13.9)	Ref		Ref		20 (15.6)	Ref		Ref		24 (13.4)	Ref		Ref	
61–65	24 (14.5)	1.07 (0.52–2.22)	0.852	0.93 (0.43–2.02)	0.860	23 (18.0)	1.18 (0.56–2.50)	0.664	1.15 (0.53–2.51)	0.731	22 (12.3)	0.94 (0.45–1.96)	0.872	0.90 (0.42–1.94)	0.793
66–70	54 (32.5)	1.12 (0.60–2.08)	0.731	0.65 (0.33–1.29)	0.220	38 (29.7)	0.90 (0.46–1.76)	0.762	0.82 (0.40–1.67)	0.578	51 (28.5)	1.01 (0.54–1.88)	0.976	0.85 (0.44–1.66)	0.634
71–75	57 (34.3)	1.33 (0.71–2.48)	0.375	0.78 (0.38–1.58)	0.490	43 (33.6)	1.15 (0.59–2.23)	0.677	0.95 (0.45–1.97)	0.883	69 (38.5)	1.54 (0.84-–2.83)	0.165	1.32 (0.68–2.58)	0.418
>75	8 (4.8)	0.94 (0.34–2.60)	0.911	0.58 (0.19–1.77)	0.339	4 (3.1)	0.54 (0.16–1.87)	0.333	0.56 (0.15–2.04)	0.379	13 (7.3)	1.47 (0.59–3.66)	0.407	1.49 (0.56–3.96)	0.427
*Sex*															
Female	92 (55.4)	1.03 (0.70–1.54)	0.867	1.06 (0.67–1.69)	0.804	67 (52.3)	0.91 (0.60–1.41)	0.682	0.89 (0.55–1.44)	0.638	96 (53.6)	0.96 (0.65–1.42)	0.847	0.97 (0.63–1.51)	0.899
*FEV*_*1*_*%pred*															
≥80	17 (10.2)	Ref		Ref		20 (15.6)	Ref		Ref		23 (12.8)	Ref		Ref	
50–79	89 (53.6)	1.70 (0.91–3.15)	0.094	1.63 (0.84–3.15)	0.145	73 (57.0)	1.18 (0.65–2.15)	0.584	1.22 (0.65–2.28)	0.539	101 (56.4)	1.42 (0.81–2.50)	0.221	1.54 (0.85–2.77)	0.156
30–49	47 (28.3)	2.89 (1.45–5.80)	0.003	2.73 (1.28–5.78)	0.009	31 (24.2)	1.62 (0.81–3.27)	0.176	1.70 (0.80–3.59)	0.168	43 (24.0)	1.96 (1.02–3.77)	0.045	2.14 (1.07–4.30)	0.032
<30	13 (7.8)	2.65 (1.02–6.84)	0.045	1.63 (0.56–4.74)	0.370	4 (3.1)	0.69 (0.20–2.39)	0.561	0.62 (0.16–2.34)	0.477	12 (6.7)	1.81 (0.71–4.59)	0.214	1.28 (0.46–3.57)	0.644
Exacerbation^a^	66 (39.8)	2.39 (1.54-3.69)	<0.001	2.64 (1.61–4.35)	<0.001	32 (25.0)	1.21 (0.73–2.00)	0.468	1.40 (0.80–2.44)	0.237	68 (38.0)	2.22 (1.44–3.41)	<0.001	2.33 (1.44–3.76)	0.001
Heart failure	29 (17.5)	2.96 (1.55–5.66)	0.001	1.93 (0.89–4.16)	0.094	13 (10.2)	1.58 (0.74–3.40)	0.240	1.54 (0.64–3.74)	0.340	20 (11.2)	1.76 (0.89–3.50)	0.107	1.40 (0.63–3.10)	0.411
IHD	34 (20.5)	1.80 (1.05–3.09)	0.031	1.49 (0.82–2.71)	0.190	20 (15.6)	1.30 (0.70–2.39)	0.406	1.16 (0.60–2.25)	0.666	28 (15.6)	1.30 (0.75–2.26)	0.358	1.15 (0.63–2.10)	0.649
AF	19 (11.4)	1.81 (0.90–3.63)	0.095	0.96 (0.42–2.19)	0.921	9 (7.0)	1.06 (0.45–2.47)	0.895	0.92 (0.35–2.41)	0.860	19 (10.6)	1.66 (0.83–3.33)	0.152	1.24 (0.55–2.79)	0.606
Hypertension	117 (70.5)	2.47 (1.62–3.75)	<0.001	2.41 (1.49–3.88)	<0.001	67 (52.3)	1.14 (0.74–1.75)	0.562	1.12 (0.69–1.83)	0.638	102 (57.0)	1.37 (0.93–2.02)	0.113	1.51 (0.97–2.34)	0.065
Stroke/TIA	18 (10.8)	1.70 (0.84–3.45)	0.139	1.48 (0.68–3-24)	0.324	6 (4.7)	0.69 (0.26–1.81)	0.448	0.61 (0.22–1.68)	0.342	15 (8.4)	1.28 (0.62–2.66)	0.508	1.06 (0.49–2.32)	0.878
Diabetes	48 (28.9)	2.03 (1.26–3.28)	0.004	1.56 (0.91–2.70)	0.109	31 (24.2)	1.60 (0.94–2.71)	0.082	1.43 (0.79–2.57)	0.236	22 (12.3)	0.70 (0.40–1.23)	0.214	0.55 (0.30–1.02)	0.057
Anxiety	26 (15.7)	0.85 (0.50–1.45)	0.552	0.60 (0.32–1.14)	0.120	15 (11.7)	0.61 (0.32–1.14)	0.123	0.55 (0.27–1.15)	0.113	28 (15.6)	0.85 (0.51–1.43)	0.540	0.73 (0.39–1.35)	0.311
Depression	41 (24.7)	1.38 (0.86–2.23)	0.183	1.61 (0.89–2.90)	0.113	22 (17.2)	0.88 (0.50–1.53)	0.641	1.25 (0.65–2.41)	0.511	41 (22.9)	1.25 (0.78–2.01)	0.351	1.65 (0.92–2.94)	0.092
Osteoporosis	13 (7.8)	1.12 (0.53–2.36)	0.777	1.04 (0.44–2.47)	0.933	13 (10.2)	1.48 (0.70-–3.16)	0.307	2.03 (0.86–4.78)	0.107	11 (6.1)	0.86 (0.39–1.88)	0.704	0.80 (0.34–1.89)	0.610
Other cancer	24 (14.5)	1.68 (0.91–3.10)	0.101	1.34 (0.68–2.65)	0.394	16 (12.5)	1.42 (0.72–2.80)	0.319	1.40 (0.68–2.89)	0.358	18 (10.1)	1.11 (0.58–2.13)	0.759	0.99 (0.50–1.98)	0.983
Asthma	15 (9.0)	0.78 (0.40–1.52)	0.472	0.71 (0.35–1.48)	0.366	13 (10.2)	0.89 (0.44–1.80)	0.748	0.82 (0.39–1.72)	0.597	18 (10.1)	0.88 (0.47–1.66)	0.696	0.84 (0.43–1.65)	0.614
*Number of comorbid conditions*^b^															
0	13 (7.8)	Ref		Ref		21 (16.4)	Ref		Ref		23 (12.8)	Ref		Ref	
1	43 (25.9)	2.86 (1.40–5.84)	0.004	3.85 (1.81–8.16)	<0.001	45 (35.2)	1.86 (0.99–3.47)	0.053	2.00 (1.03–3.86)	0.040	59 (33.0)	2.22 (1.22–4.03)	0.009	2.50 (1.33–4.69)	0.005
2	41 (24.7)	3.27 (1.58–6.74)	0.001	3.69 (1.71–7.95)	0.001	29 (22.7)	1.43 (0.73–2.80)	0.296	1.52 (0.74–3.12)	0.250	48 (26.8)	2.16 (1.17–4.01)	0.014	2.18 (1.13–4.21)	0.021
≥3	69 (41.6)	5.22 (2.61–10.45)	<0.001	5.63 (2.68–11.79)	<0.001	33 (25.8)	1.55 (0.80–2.98)	0.194	1.67 (0.83–3.37)	0.155	49 (27.4)	2.09 (1.13–3.87)	0.018	2.11 (1.09–4.06)	0.026

Multinomial logistic regression with the type of check-up (no check-up, check-up only by GP, check-up only by COPD nurse, or check-up by both GP and nurse) as the dependent variable, and no check-up as the reference category.

*GP* general practitioner, *FEV*_*1*_*%pred* FEV_1_% of predicted, *IHD* ischaemic heart disease, *AF* atrial fibrillation, *TIA* transient ischaemic attack, *RRR* relative risk ratio, *CI* confidence interval.

^a^Exacerbation ≥1 previous 6 months.

^b^Separate model without individual diseases.

There were no statistically significant associations between having check-ups by the COPD nurse only and individual comorbid conditions or exacerbations. When using the comorbidity count instead of individual diseases, there was a positive association with having one comorbid condition (no comorbidity as the reference category) but the association did not remain statistically significant for a larger number of comorbid conditions.

There was a statistically significant association between having routine check-ups both by the GP and the COPD nurse and exacerbations. There was a positive association with depression (RRR 1.65, 95% CI 0.92–2.94) and an inverse association with diabetes (RRR 0.55, 95% CI 0.30–1.02), although neither of these was statistically significant. When using the comorbidity count in the model, statistically significant associations with risk ratios above 2.00 for all groups (1, 2 and ≥3 comorbid conditions) were found.

To further evaluate de-prioritisation of COPD, logistic regression analysis examined associations with having check-ups by a GP regardless of visits to a COPD nurse. The adjusted analysis showed statistically significant associations between having exacerbations or hypertension and consultations in which COPD was discussed (see Table 3). When a comorbidity count was used, a higher count was statistically significantly associated with having routine check-ups by a GP, with odds ratios ranging from 2.34 to 2.81, with no comorbidity used as the reference category. Some 48% of the patients had at least one check-up by a GP during the previous two years.

**Table 3:** Factors associated with having check-ups by a general practitioner.

	*N* (%)	Unadjusted		Adjusted	
		OR (95% CI)	*p*-value	OR (95% CI)	*p*-value
Exacerbation^a^	134 (38.8)	2.15 (1.55–2.97)	<0.001	2.16 (1.50–3.10)	<0.001
Heart failure	49 (14.2)	1.94 (1.19–3.14)	0.008	1.41 (0.79–2.49)	0.244
IHD	62 (18.0)	1.39 (0.93–2.09)	0.109	1.22 (0.78–1.91)	0.381
Atrial fibrillation	38 (11.0)	1.70 (1.00–2.88)	0.049	1.14 (0.62–2.11)	0.675
Hypertension	219 (63.5)	1.72 (1.27–2.32)	<0.001	1.79 (1.27–2.52)	0.001
Stroke/TIA	33 (9.6)	1.66 (0.95–2.91)	0.075	1.52 (0.83–2.77)	0.176
Diabetes	70 (20.3)	1.07 (0.74–1.54)	0.739	0.85 (0.56–1.28)	0.428
Anxiety	54 (15.7)	0.99 (0.66–1.49)	0.968	0.80 (0.50–1.30)	0.365
Depression	82 (23.8)	1.38 (0.96–1.98)	0.084	1.49 (0.96–2.31)	0.076
Osteoporosis	24 (7.0)	0.84 (0.48–1.47)	0.547	0.71 (0.38–1.33)	0.284
Other cancer	42 (12.2)	1.20 (0.76–1.92)	0.435	1.04 (0.63–1.73)	0.865
Asthma	33 (9.6)	0.87 (0.53–1.41)	0.566	0.84 (0.50–1.43)	0.526
*Number of comorbid conditions*^b^					
0	36 (10.4)	Ref		Ref	
1	102 (29.6)	2.00 (1.24–3.22)	0.004	2.34 (1.42–3.87)	0.001
2	89 (25.8)	2.30 (1.40–3.76)	0.001	2.35 (1.39–3.97)	0.001
≥3	118 (34.2)	2.82 (1.74–4.55)	<0.001	2.81 (1.68–4.68)	<0.001

Logistic regression with check-ups by a general practitioner as dependent variable. Adjusted for age, sex, BMI, county council and FEV_1_% of predicted.

*IHD* ischaemic heart disease, *TIA* transient ischaemic attack, *OR* odds ratio, *CI* confidence interval, *BMI* Body Mass Index.

^a^Exacerbation ≥ 1 previous 6 months.

^b^Separate model without individual diseases.

The results from the analysis of health care centre characteristics are shown in Table 4. Most of the patients attended a PHCC with a nurse-led COPD clinic (85%) which showed a positive association with having check-ups by a COPD nurse or both nurse and GP, and an inverse association with having check-ups *only* by a GP. With an increasing number of registered patients at the PHCC, it was less likely for the patients to have check-ups only by a GP and more likely to have check-ups only by a COPD nurse or by both GP and nurse. There were no statistically significant associations with whether the PHCC being privately managed or not. Associations between check-ups and the proportion of permanently employed GPs at the health care centre, were inconsistent.

**Table 4:** Association between structural factors at the health care centre and check-ups.

*N* = 660	Check-up only by GP, *n* = 159 (24.1%)					Check-up only by COPD nurse, *n* = 116 (17.6%)					Check-up by both GP and COPD nurse, *n* = 167 (25.3%)				
	*N*^a^ (%)	Unadjusted		Adjusted		*N*^a^ (%)	Unadjusted		Adjusted		*N*^a^ (%)	Unadjusted		Adjusted	
		RRR (95% CI)	*p*-value	RRR (95% CI)	*p*-value		RRR (95% CI)	*p*-value	RRR (95% CI)	*p*-value		RRR (95% CI)	*p*-value	RRR (95% CI)	*p*-value
*Registered patients at PHCC*															
≤5000	35 (22.0)	Ref		Ref		13 (11.2)	Ref		Ref		17 (10.2)	Ref		Ref	
5001–10,000	70 (44.0)	0.92 (0.52–1.60)	0.757	0.97 (0.48–1.96)	0.923	45 (38.8)	1.59 (0.77–3.28)	0.214	1.30 (0.55–3.07)	0.557	66 (39.5)	1.78 (0.92–3.43)	0.086	1.95 (0.92–4.14)	0.080
10,001–15,000	42 (26.4)	0.70 (0.39–1.28)	0.248	0.70 (0.35–1.41)	0.322	33 (28.4)	1.48 (0.70–3.16)	0.306	1.31 (0.56–3.05)	0.529	48 (28.7)	1.65 (0.83–3.27)	0.150	2.18 (1.02–4.67)	0.044
>15,000	12 (7.5)	0.41 (0.18–0.91)	0.029	0.47 (0.18–1.22)	0.122	25 (21.6)	2.28 (1.01–5.18)	0.048	1.56 (0.61–4.00)	0.351	36 (21.6)	2.52 (1.20–5.29)	0.015	2.10 (0.90–4.92)	0.087
*Proportion permanently employed GPs*															
≥75%	71 (44.7)	Ref		Ref		42 (36.2)	Ref		Ref		68 (40.7)	Ref		Ref	
50–74%	51 (32.1)	1.01 (0.63–1.63)	0.954	0.79 (0.43–1.46)	0.451	33 (28.4)	1.65 (0.98–2.76)	0.058	2.14 (1.05–4.36)	0.036	64 (38.3)	1.33 (0.84–2.11)	0.227	1.64 (0.90–2.97)	0.107
<50%	37 (23.3)	0.93 (0.55–1.56)	0.773	1.03 (0.50–2.10)	0.939	25 (21.6)	1.06 (0.58–1.92)	0.853	0.86 (0.41–1.84)	0.704	35 (21.0)	0.92 (0.54–1.55)	0.741	0.81 (0.42–1.56)	0.526
Presence of COPD clinic	117 (73.6)	0.57 (0.35–0.94)	0.027	0.50 (0.27–0.93)	0.029	111 (95.7)	4.54 (1.73–11.89)	0.002	5.70 (1.98–16.38)	0.001	150 (89.8)	1.80 (0.98–3.33)	0.060	1.93 (0.95–3.92)	0.071
County council	133 (83.6)	1.08 (0.63–1.87)	0.783	1.56 (0.75–3.27)	0.238	105 (90.5)	2.02 (0.99–4.11)	0.054	0.56 (0.20–1.54)	0.263	148 (88.6)	1.64 (0.91-2.97)	0.100	0.93 (0.42–2.06)	0.849
Private	26 (16.4)	Ref		Ref		5 (4.3)	Ref		Ref		19 (11.4)	Ref		Ref	

Multinomial logistic regression with type of check-up (no check-up, check-up only by GP, check-up only by COPD nurse, or check-up by both GP and nurse) as dependent variable, and no check-up as reference category. Variables added to model with individual diseases and potential confounding factors of age, sex, BMI, county council, and FEV_1_% of predicted.

*GP* general practitioner, *PHCC* primary health care centre, *BMI* body mass index, *RRR* relative risk ratio, *CI* confidence interval.

^a^Number of patients.

## Discussion

The first main finding of this study was that COPD patients with multimorbidity tend to see the general practitioner and COPD nurse more often for COPD check-ups than those without multimorbidity. Our results are not consistent with de-prioritisation of COPD because of multimorbidity. However, we do not know the amount of time devoted to COPD during the consultations or the quality of disease management. The second main finding was that having exacerbations was positively associated with having routine COPD check-ups by the GP.

COPD patients with a higher number of comorbid diseases were more likely to have routine check-ups by the GP, or by both GP and COPD nurse, for monitoring of COPD. Patients with multimorbidity were more frequent care users, potentially resulting in surveillance bias such that there are more opportunities to make other diagnoses, such as hypertension (although all patients with COPD will have their blood pressure tested), which may be more likely recorded due to frequent testing, thus inflating the total number of diagnoses. More visits to a GP due to multimorbidity increases the opportunity to discuss COPD and this could in part explain the higher magnitude association of multimorbidity with COPD being mentioned. This is mainly relevant for GP visits and not COPD nurse visits, where we did not identify evidence of de-prioritisation.

As in previous studies, comorbidity was common in COPD patients^7,8^. Of the individual comorbid diseases, only hypertension was statistically significantly associated with receiving more frequent routine check-ups for COPD. Hypertension is likely to be the most common comorbidity in COPD patients^1^, as here 57% of the patients had the diagnosis. Although hypertension is common in the general population, it appears to occur even more frequently in COPD patients^9^. Hypertension is associated with the increased systemic inflammation in COPD^10^ but has not been found to increase mortality^11^ or risk of exacerbations^7^. However, it is an important risk factor for other cardiovascular diseases.

We found a positive, but not statistically significant, association between depression and having routine check-ups. Some 21% of the patients in the study had a diagnosis of depression in their medical records sometime during the study period. COPD has been found to increase the risk of developing depression, and comorbid depression has been associated with an increased risk of exacerbations and mortality in COPD patients^12^. Many patients have had persistent depressive symptoms for several years^13^.

COPD patients with comorbid diabetes have been found to have an increased risk of severe exacerbations^14^. In our study, a positive association was found between diabetes and having check-ups only by a GP or a COPD nurse, but an inverse for having check-ups by both. One possible explanation for this could be that patients with diabetes often have annual check-ups by a specialised diabetes nurse, which may result in fewer check-ups by a COPD nurse. The logistic regression analysis indicated inconclusively that patients with diabetes may have a somewhat reduced number of check-ups by a GP, when not considered mutually exclusive, as in the multinomial models. It remains possible to speculate that the pattern of management is different for these patients in a way that de-prioritises COPD over diabetes: a Danish primary care study, found that patients with coexisting COPD and diabetes had annual control visits for their COPD less frequently than patients with only COPD^15^.

At least one exacerbation during the previous 6 months, was positively associated with having routine check-ups of COPD by the GP or by both GP and COPD nurse. Having a history of exacerbations is recognised to be an important risk factor for having additional exacerbations^16,17^ and for deterioration of the disease^1^. Several comorbid conditions are also associated with an increased risk of exacerbations, such as heart disease^3^ and asthma^18^. The focus of long-term COPD management is recommended to be on symptom relief and preventing future exacerbations^1^. The results of this study suggest that primary healthcare is giving more attention to COPD management if patients have had more frequent exacerbations, which complies with this recommendation.

More than half of the patients in the study did not have any routine check-ups by the GP during the previous 2 years. One-third did not have check-ups by a GP or nurse. Some of these patients may have had COPD check-ups in secondary care, but as this would only add to the number of consultations it would provide no additional evidence of COPD de-prioritisation in primary care. Patients without check-ups in primary care had less comorbidity, less frequent exacerbations, and less severe air-flow limitation. This could be seen as an example of giving care according to need in a primary healthcare setting struggling to keep up with the rising demands of an aging population. In healthier individuals with less severe COPD disease outcomes, COPD received less attention. The future consequences of this omission of routine check-ups for less severe COPD requires further research.

In a 2016 qualitative study in Sweden, GPs were interviewed about their views on the management of COPD in multimorbid patients^5^. They stated that COPD was less likely to be discussed during check-ups if the patient had few symptoms of COPD, whereas a history of recurrent respiratory infections or obvious airway symptoms would most likely lead to a discussion about COPD. This is consistent with our finding that patients with exacerbations were more likely to have routine check-ups by the GP than those without exacerbations. However, in contrast with this qualitative study, we could not confirm any de-prioritisation of COPD because of multimorbidity in our study population.

As we identified routine check-ups by the GP through a text reference to COPD symptoms or management in the medical records, we did not have any information about how time was divided between COPD and the patient’s other chronic diseases. Hence, we could not assess the prioritisation of COPD relative to other chronic diseases. We defined de-prioritisation as not having at least one COPD-related check-up by a GP during a 2-year period, as this is recommended by national guidelines. However, having one check-up does not indicate intensive management of COPD. Neither were we able to separate previously planned visits from those when patients sought care for another reason and also discussed COPD at this time.

Having check-ups by a COPD nurse was not statistically significantly associated with comorbidity, exacerbations, or lung function, but there were statistically significant differences between the county councils that were included in the study. This may be because of differences in local procedures, and patients attending the nurse-led COPD clinic specifically because of a COPD diagnosis, in contrast with a check-up by the GP that could involve managing several conditions during a single consultation.

The shortage of permanently employed GPs and its consequences is an often-discussed question in primary health care in Sweden. In this study, we could not find consistent associations between staffing problems and how COPD was managed at the PHCCs. Nor could we find any association with whether they were private or operated by the county council. In larger centres, there was a greater likelihood for patients receiving check-ups by both GP and nurse. Presence of asthma/COPD clinics, which most centres had, significantly increased the chances of having check-ups by a COPD nurse.

Adherence to guidelines is still often used to measure the quality of care^19^. In multimorbid patients a holistic approach is preferable, and clinical judgement becomes more important in GPs’ work when the patient’s individual needs are not well-served by single-disease guidelines^20^. During some consultations, one condition might receive less attention than another, based upon the patient’s current needs and preferences and the doctor’s and patient’s prioritisation of them. The NICE guidelines for multimorbidity aim to improve quality of life by shared decision-making^2^. Hence, routine check-ups of multimorbid COPD patients do not necessarily involve reviewing all conditions every time.

A strength of this study was the sample size of over 700 patients with a doctor’s diagnosis of COPD, randomly selected from 76 primary health care centres providing real-world data from clinical practice with good external validity.

The use of both medical records and self-completion questionnaires was a strength, allowing for information about the patient’s own experience of symptoms to be combined with information from records. Data on exacerbations came from self-completion questionnaires and was dependent on patients’ recollection, and therefore a time period of the previous 6 months was chosen.

A limitation of the study was that not all patients had spirometry data in their records, or data on exacerbations and BMI in their questionnaires, which reduced the number of patients included in the analysis. The excluded patients had similar distributions for age, sex, and exacerbations, compared to the patients with spirometry data.

Some 59% completed the questionnaire. The non-respondents were slightly younger and more often women. Thus, the study participants tended to be older, and thus more likely to have a comorbidity, which may have affected the results.

Among the 76 PHCCs, 71 (93%) completed the questionnaire about organisational characteristics, somewhat reducing the number of patients included in this analysis.

Another limitation was the limited number of comorbid diseases included in the analyses, but they were chosen on a theoretical basis and should illustrate the association. Given the direction of association with priority, we do not think that adding more diseases would reverse this. A previous study found that increasing the number of conditions considered, increased the prevalence of multimorbidity^21^.

The comorbid conditions were noted as present or not in the record review, during or before the studied period. We have not considered their severity or duration. This is of potential concern, especially for depression as it is not necessarily a long-term diagnosis even though COPD patients often have persistent symptoms of depression^13^.

An alternative interpretation of the findings of more frequently mentioned COPD during consultations among those with multimorbidity is that there is de-prioritisation of COPD treatment—or other influences—such that worse disease characteristics result in more frequent mentions of COPD. To address this concern, we adjusted for both lung function and COPD exacerbations. This made little difference to the results, but as the measurement of exacerbations was not a particularly fine grain (at least once during the previous 6 months), there is a possibility of residual confounding.

A limitation to the validity of our data may have been the occasions when COPD was assessed but the assessment was not described in the medical records, and therefore not detected in the review. Also, we do not know how much time was spent on COPD during the check-up, if the patients themselves brought up COPD, or what the consultation resulted in.

COPD patients in primary care more often had routine check-ups if they had greater disease severity, multimorbidity, or frequent exacerbations, suggesting that those in need of attention received it. However, we do not know the extent or quality of the consultation time devoted to COPD, justifying further research into the quality of COPD management in patients with comorbid diseases.

## Methods

### Design and study population

The patient cohort in this study consisted of the second PRAXIS COPD cohort that was created in 2014 by recruiting patients from 76 primary health care centres in seven county councils in central Sweden^22^. Of 2156 randomly selected patients, with the ICD-code J44 in their medical records between 2007 and 2010, 1267 (59%) completed a self-completion questionnaire in 2014 and 1190 patients approved medical record reviews. Of these, 1163 records were retrieved and reviewed (see Fig. 1).

### Data collection and measures

The medical record reviews were carried out by two research nurses in accordance with a detailed template with support from the PRAXIS research group, to standardise assessment. The records were from 2004 to 2014, and the individual period covered varied between 2 and 10 years. Comorbid diseases were identified either by the ICD code or as free text in the medical records. The 11 comorbid conditions with the highest frequency were included in the analysis (see Table 1). Cancer was identified as lung cancer and other cancers were grouped together. Depression and anxiety were defined as having the diagnosis sometime during the period of record review. Type 1 and 2 diabetes were considered together. The record review ensured that patients whose COPD diagnosis had been removed during the study period were not included in the analysis.

Data on routine check-ups by a GP where COPD was assessed, during a two-year period with follow-up to 2014, were extracted. For a check-up to be included there needed to be a text reference to COPD symptoms or management in the notes. Consultations where only prescriptions were renewed, or the diagnostic code for COPD (J44) was registered without any text reference, were excluded. The two-year period was chosen for consistency with the questionnaire that was completed in 2014. Data on routine check-ups with the COPD nurse during the same time period were extracted. Unscheduled/emergency visits to a COPD nurse or GP, due to worsening COPD symptoms, were not included. De-prioritisation of COPD was defined as not receiving routine check-ups by a GP, where COPD was noted in the medical record, at least once during the two-year period. The Swedish National Board of Health and Welfare recommends annual check-ups of COPD patients with maintenance treatment^23^.

The questionnaire collected information on height and weight, and frequency of exacerbations during the previous 6 months. BMI was calculated from questionnaire data. An exacerbation was defined as an unscheduled/emergency visit to primary or secondary care, and/or use of oral steroids, and/or use of antibiotics due to worsening of COPD symptoms, in the previous six months.

Most of the patients’ records had data on forced expiratory volume in one second (FEV_1_) pre- or post-bronchodilator, expressed as a percentage of the predicted value (FEV_1_%pred) from diagnostic or more recently performed spirometry. Where several values were available, the highest FEV_1_%pred was selected. FEV_1_% of predicted was used as a measure of COPD severity.

In 2012, the PHCCs in the study responded to a questionnaire about a number of registered patients at the PHCCs or persons in the catchment area, the proportion of permanently employed GPs of the total number of posts, and if they had a nurse-led COPD clinic.

### Statistical analysis

Analyses were performed using SPSS version 26.0 (SPSS, Chicago). Cross-tabulation and the chi-square tests were used to examine differences in age, sex, BMI, exacerbations, and comorbid conditions between the group with at least one routine check-up by a GP, COPD nurse, or both where COPD was assessed, and the group with no such check-ups during the previous two years. All measures were modelled as categorical variables.

The associations between having routine check-ups and comorbid diseases and exacerbations were analysed using multinomial regression analysis with check-ups (no check-up, only with GP, only with COPD nurse, or both) as the dependent variable and the 11 most frequent comorbid diseases, presence of at least one exacerbation during the previous 6 months and the potential confounding factors of age, sex, BMI, FEV_1_% of predicted, and county council as independent variables. Having had no check-ups was used as the reference category. The analysis was repeated with the summarised number of comorbid conditions (0, 1, 2 or ≥3) as an independent variable instead of the individual comorbid diseases. Relative risk ratios with 95% confidence intervals were calculated.

To further evaluate de-prioritisation, we used logistic regression analysis with having at least one routine check-up by the GP during the previous two years, regardless of check-ups by the nurse, as the dependent variable, and comorbid diseases, exacerbations, age, sex, BMI, FEV_1_% of predicted, and county council as independent variables. Odds ratios (OR) with 95% confidence intervals were calculated. The analysis was repeated with the summarised number of comorbid conditions (0, 1, 2 or ≥3) as an independent variable.

For the subgroup of PHCCs with data about organisational characteristics, an additional multinomial regression analysis was performed. The total number of registered patients, proportion of permanently employed GPs, presence of a nurse-led COPD clinic, and if the health care centre was run by the county council or a private company, were included in the analysis.

A *p*-value < 0.05 or confidence intervals not including 1.00 were considered statistically significant.

### Ethics

The study was approved by the Regional Ethical Board in Uppsala, Sweden (DNR 2011/318). All participants gave written informed consent.

### Reporting summary

Further information on research design is available in the Nature Research Reporting Summary linked to this article.

## Supplementary information

REPORTING SUMMARY

## Data Availability

The data that support the findings of this study are available from Björn Ställberg (b.stallberg@telia.com), upon reasonable request with appropriate ethical permission.
